# Potentiation of amyloid beta phagocytosis and amelioration of synaptic dysfunction upon FAAH deletion in a mouse model of Alzheimer's disease

**DOI:** 10.1186/s12974-021-02276-y

**Published:** 2021-09-29

**Authors:** Gonzalo Ruiz-Pérez, Samuel Ruiz de Martín Esteban, Sharai Marqués, Noelia Aparicio, M. Teresa Grande, Irene Benito-Cuesta, Ana M. Martínez-Relimpio, M. Andrea Arnanz, Rosa M. Tolón, María Posada-Ayala, Benjamin F. Cravatt, José A. Esteban, Julián Romero, Rocío Palenzuela

**Affiliations:** 1 Faculty of Experimental Sciences, Universidad Francisco de Vitoria, Pozuelo de Alarcón, 28223 Madrid, Spain; 2 The Skaggs Institute for Chemical Biology and Departments of Cell Biology and Chemistry, The Scripps Research Institute, La Jolla, San Diego, CA 92037 USA; 3 Centro de Biología Molecular Severo Ochoa, CSIC-Universidad Autónoma de Madrid, 28049 Madrid, Spain

**Keywords:** Alzheimer’s disease, Anandamide, Hippocampal synaptic plasticity, Dendritic spines, TREM2, Complement

## Abstract

**Background:**

The complex pathophysiology of Alzheimer’s disease (AD) hampers the development of effective treatments. Attempts to prevent neurodegeneration in AD have failed so far, highlighting the need for further clarification of the underlying cellular and molecular mechanisms. Neuroinflammation seems to play a crucial role in disease progression, although its specific contribution to AD pathogenesis remains elusive. We have previously shown that the modulation of the endocannabinoid system (ECS) renders beneficial effects in a context of amyloidosis, which triggers neuroinflammation. In the 5xFAD model, the genetic inactivation of the enzyme that degrades anandamide (AEA), the fatty acid amide hydrolase (FAAH), was associated with a significant amelioration of the memory deficit.

**Methods:**

In this work, we use electrophysiology, flow cytometry and molecular analysis to evaluate the cellular and molecular mechanisms underlying the improvement associated to the increased endocannabinoid tone in the 5xFAD mouse^−^ model.

**Results:**

We demonstrate that the chronic enhancement of the endocannabinoid tone rescues hippocampal synaptic plasticity in the 5xFAD mouse model. At the CA3–CA1 synapse, both basal synaptic transmission and long-term potentiation (LTP) of synaptic transmission are normalized upon FAAH genetic inactivation, in a CB1 receptor (CB1R)- and TRPV1 receptor-independent manner. Dendritic spine density in CA1 pyramidal neurons, which is notably decreased in 6-month-old 5xFAD animals, is also restored. Importantly, we reveal that the expression of microglial factors linked to phagocytic activity, such as TREM2 and CTSD, and other factors related to amyloid beta clearance and involved in neuron–glia crosstalk, such as complement component C3 and complement receptor C3AR, are specifically upregulated in 5xFAD/FAAH^−/−^ animals.

**Conclusion:**

In summary, our findings support the therapeutic potential of modulating, rather than suppressing, neuroinflammation in Alzheimer’s disease. In our model, the long-term enhancement of the endocannabinoid tone triggered augmented microglial activation and amyloid beta phagocytosis, and a consequent reversal in the neuronal phenotype associated to the disease.

**Supplementary Information:**

The online version contains supplementary material available at 10.1186/s12974-021-02276-y.

## Background

Alzheimer’s disease (AD) is a devastating neurodegenerative disorder that threatens to impose an unbearable social and economic burden in the years to come due to the lack of effective treatments [1]. It entails a complex pathophysiological scenario, with progressive incapacitating dementia as its main hallmark. The major neuropathological events include the extracellular deposition of amyloid-β (Aβ) peptides in plaques, the intraneuronal accumulation of hyperphosphorylated tau protein in neurofibrillary tangles, and the synapse dysfunction that precedes neuronal loss [2]. Glial activation and neuroinflammation result from the accumulation of aberrant proteins, although it is still unclear if these factors contribute to worsen the pathological scenario, or otherwise halt disease progression [3]. Whether glia promotes neurodegeneration or exerts a neuroprotective effect in AD is currently under intense debate.

Neuroinflammation is usually defined as a “double-edged sword”, as its specific consequences, either beneficial or detrimental, seem to intimately depend on the context in which it is taking place. In addition, it appears to be crucially influenced by neuron–glia interaction. Microglial cells play a key role in sensing tissue homeostasis and can respond to a changing neuron environment, especially in pathological conditions [4]. Astrocytes display a previously unknown plasticity, as their functions (essential for normal neuronal development, synapse formation and propagation of action potentials) seemingly change in response to brain damage [5]. A third layer of complexity is added by the bidirectional communication between astrocytes and microglia, that shapes their reactivity and ability to respond to a varied range of stimuli in the CNS [6].

In this context, numerous reports point to the delicate equilibrium of the neuron–glia crosstalk in neurodegenerative diseases, that in turn determines the effects of neuroinflammation. Although originally thought to be detrimental for AD, gliosis and neuroinflammation could have neuroprotective roles early in AD, to become toxic to neurons afterwards, by promoting neurodegeneration. Consequently, the regulation of inflammatory responses, rather than their suppression, has been proposed as a therapeutic strategy in neurodegenerative diseases [3, 7]. In turn, the modulation of the endocannabinoid system (ECS), composed of a wide array of receptors and endogenous ligands, such as anandamide (AEA), could be instrumental to fulfill this therapeutic goal [8, 9].

We previously reported the beneficial impact of neuroinflammation on AD, using a mouse model of the disease that expresses five human familial AD gene mutations, the 5xFAD. The genetic inactivation of fatty acid amide hydrolase (FAAH), the enzyme that degrades anandamide (AEA), among other N-acylethanolamines, was associated with the induction of a pro-inflammatory milieu in 5xFAD animals, and yet, with a significant cognitive rescue [10]. The hippocampal-dependent memory deficit that is defining of this aggressive model of amyloidosis [11], was reversed in 5xFAD/FAAH^−/−^ animals, together with a reduction in amyloid peptide levels and plaque burden. Paradoxically, the activation of microglia cells was enhanced comparing to 5xFAD mice [10]. These observations matched those obtained in another mouse model of acute insult to the brain [12].

As mentioned before, the modulation of the endocannabinoid system has long been envisioned as a promising therapeutic strategy in human disorders in which the neuroinflammatory component plays a central part, such as AD [8, 9]. Our previous work, revealing a phenotypic rescue in 5xFAD animals upon the long-term enhancement of the endocannabinoid tone, clearly pointed in this direction. Nevertheless, a comprehensive characterization of the molecular mechanisms involved this endocannabinoid-mediated cognitive improvement was still lacking. In this work, we aimed to uncover the underlying cellular processes responsible for the beneficial effects on neuropathology resulting from FAAH genetic inactivation.

## Methods

### Mice

Mice used in these experiments were described in our previous reports [10, 13]. Briefly, 5xFAD mice, coexpressing 3 Familiar Alzheimer’s Disease (FAD) mutations in the human amyloid precursor protein (APP) gene and 2 FAD mutations in the human presenilin 1 (PS1) gene, were purchased from Jackson Laboratories (Bar Harbor, ME, USA, [11]). Mice with deletion of the gene for FAAH (FAAH^−/−^ mice, [14]) in the C57BL/6 J background were mated with 5xFAD mice and backcrossed for at least 10 generations to generate paired 5xFAD and 5xFAD/FAAH^−/−^ littermates. Mice were housed and bred in the animal facilities of Universidad Francisco de Vitoria (Pozuelo de Alarcón, Madrid, Spain). The experimental protocols met the European and Spanish regulations for protection of experimental animals (89/609/EEC and RD 1201/2005 and 53/2013).

### Electrophysiology

Acute hippocampal slices of 300 µm-thickness were obtained from 6-month-old WT, WT/FAAH^−/−^, 5xFAD and 5xFAD/FAAH^−/−^ littermates and maintained in artificial cerebrospinal fluid (ACSF), gassed with 95% O_2_/5% CO_2_, at room temperature. ACSF consisted of 119 mM NaCl, 2.5 mM KCl, 26 mM NaHCO_3_, 1 mM NaH_2_PO_4_, 11 mM glucose, 2.5 mM CaCl_2_, 1.2 mM MgCl_2_. pH was adjusted to 7.4 and osmolarity to 290 mOs. After 60 min recovery (at 33 °C), slices were placed in the recording chamber.

Synaptic responses were evoked with bipolar electrodes using single-voltage pulses. The stimulating electrodes were placed over Schaffer collateral fibres in *stratum radiatum* area. The field excitatory postsynaptic potentials (fEPSPs) were recorded with glass electrodes (filled with ACSF) placed in the apical dendritic layer of CA1 area. Recordings were obtained with Multiclamp 700 A/B amplifiers and analysed with pClamp software (Molecular Devices).

Experiments were carried out in the presence of picrotoxin (100 µM) at 25 °C. In long-term potentiation (LTP) and long-term depression (LTD) experiments, baseline stimulation was maintained for at least 20 min before induction. Responses were recorded up to 1 h after the induction. LTP was induced by theta-burst stimulation (4 trains of 10 bursts at 5 Hz, each burst consisting of 4 pulses at 100 Hz). NMDAR-dependent LTD was induced with 1 Hz, 900 pulses Schaffer collateral stimulation. Paired-pulse facilitation (PPF) was elicited using increasing inter-stimulus intervals (50, 100, 200 and 400 ms).

### Pharmacological treatments

In some LTP experiments, a CB1R antagonist (AM251, at 4 µM; Tocris Bioscience) and a TRPV1 antagonist (AMG9810 at 3 µM; Tocris Bioscience) were used, with DMSO at 14 mM as vehicle. The dosing concentration for AM251 and AMG9810 was chosen according to previous reports [15, 16]. Drugs or vehicle were added to the bath prior to electrophysiological recordings and to circulating ACSF during fEPSPs recordings. Incubation with the drugs or vehicle was maintained for at least 20 min before starting recordings.

### DiOlistic labelling

6-month-old animals were deeply anesthetized by i.p. injection of a mixture of ketamine (170 mg/kg) and xylazine (10.7 mg/kg) and transcardially perfused with PBS 25 mM, pH 7.5 followed by fixative solution (4% paraformaldehyde and 4% sucrose in PBS 25 mM, pH 7.5). Brain was removed and postfixed 10 min in fixative solution before cutting on vibratome (Leica VT 1000 S) to obtain 200 µm-thickness slices, maintained in PBS. Before shooting slices, tungsten microcarriers (1.3 µm diameter, Bio-Rad) were coated with the lipophilic carbocyanine dye DiI (Invitrogen, D3911) in a ratio of 3 mg of DiI per 100 mg of Tungsten particles. DiI-coated particles were delivered diolistically into the slices using a gene gun apparatus (Helios Gene Gun System, Bio-Rad). Slices were stored 24 h in the dark at room temperature allowing DiI to diffuse throughout the membranes. After incubation, slices were postfixed with fixative solution at room temperature and finally mounted onto superfrost glass slides with ProLong Gold Antifade reagent (Invitrogen, P36930).

### Morphological analysis of dendritic spines

Fluorescence images of CA1 pyramidal neurons were obtained with an upright microscope (Axiovert Imager.Z1M, Zeiss) coupled to a confocal laser scanning microscope LSM510 using a 63 × oil immersion objective. For quantification, only apical secondary dendrites 100–200 µm distal to the soma from fully stained CA1 pyramidal neurons were used. Confocal z stacks were taken every 0.14 μm at 1024 × 1024 pixel resolution and were deconvolved using the Huygens software (Scientific Volume Imaging) to reduce optical aberration along the z axis. The total number of spines counting, and their morphology categorization were performed using the Vias and NeuronStudio freeware (NeuronStudio, RRID:SCR_013798). Quantification of spine types was carried out as previously described [17, 18]. Essentially, spines were automatically classified in three categories (stubby, thin, or mushroom) depending on the head to neck ratio (“neck ratio”), the length to head diameter ratio (“thin ratio”) and the head diameter value (0.35 µm). Spines with a < 1.1 or > 1.1 neck ratio, a thin ratio < 2.5 and a head diameter < 0.35 µm were classified as stubby spines. Spines with a neck ratio > 1.1 and a thin ratio > 2.5 were categorized as thin spines. Spines with a neck ratio > 1.1 and a head diameter ≥ 0.35 µm were classified as mushroom spines. From these measurements, the percentage of the various spine types was calculated. At least 21 dendrites per group from a minimum of 3 different neurons and at least 3 animals per genotype were counted. Image analysis was performed independently by two investigators blind to genotypes and results were then pooled.

### Isolation of microglial cells and flow cytometry

Ability of microglial cells to phagocytose amyloid beta was measured by flow cytometry. 6-month-old animals were i.p. injected with Methoxy X04 (Tocris Bioscience) at 10 mg/kg body weight. 24 h after injection, animals were deeply anesthetized by i.p. administration of a mixture of ketamine (170 mg/kg) and xylazine (10.7 mg/kg) and transcardially perfused with PBS 1X, pH 7.5. Brains were dissected and enzymatically digested to facilitate microglia separation. The cell suspension was mechanically dissociated and filtered through a 70 µm-cell strainer. Microglial cells, isolated by percoll gradient, were washed with PBS 1X and blocked with 0.3% BSA/PBS 1X for 20 min. Cells were stained with CD11b-PE and CD45-APC antibodies (Miltenyi Biotec) for 40 min. Samples were read on a MACSQuant Flow Cytometer and analysed with MACS Quantify software (Miltenyi Biotec).

Debris and aggregates were eliminated from analysis by forward and side scatter characteristics, then microglia were identified as CD11b^+^ CD45^lo^. Fluorescence signals were corrected by fluorescence minus one (FMO) controls. For each hemisphere, approximately ten thousand CD11b^+^ singlets were analysed.

### Quantification of protein expression by reverse-transcriptase polymerase chain reaction (RT-qPCR)

Mice were sacrificed by cervical dislocation and brains quickly extracted. Only the hippocampal area was used for these experiments. Tripure Isolation Reagent (Roche Diagnostics) was used to isolate total RNA for real-time PCR assays, according to the manufacturer’s instructions. RNA was dissolved in RNase-free water, and its concentration was quantified by absorption at 260 nm. Single-stranded complementary DNA (cDNA) was synthesized from 1 µg of total RNA using the Transcriptor First Strand cDNA Synthesis Kit (Roche). Gene expression was quantified by real-time PCR in a CFX Connect® Real-Time PCR Detection System (Bio-Rad), using either the LightCycler FastStart DNA Master HybProbe (Roche), for 18S primers and probe number 55 from Universal ProbeLibrary (Roche), chosen for normalization, or the PrimePCR™ primers and probe assays from Bio-Rad, for the genes of interest (CD68, qMmuCEP0027967; TREM2, qMmuCEP0054571; CD33, qMmuCIP0029386; CX3CR1, qMmuCEP0058111; CD200R, qMmuCIP0036671; APOE, qMmuCEP0053239; CLEC7A, qMmuCEP0059955; CTSD, qMmuCIP0032405; P2RY12, qMmuCEP0057087; TMEM119, qMmuCEP0042925; C1QA, qMmuCEP0028019; C1QB, qMmuCEP0057436; C1QC, qMmuCEP0057437; C3, qMmuCEP0054671; C3AR, qMmuCEP0056708; C3R (ITGAM), qMmuCIP0030148). The Quantimix Easy Probes Kit from Biotools was used for amplification. All assays were carried out twice as independent PCR runs for each cDNA sample. Mean values were used for further calculation. A negative (no template) control was measured in each of the PCR runs. To obtain the Relative Quantification (RQ) values, used for statistical analyses, the Cq values were analysed with the 2^−ΔΔCt^ algorithm.

### Quantification of TREM2 by immunofluorescence

Six-month-old 5xFAD and 5xFAD/FAAH^−/−^ mice (*n* = 3 per group) were deeply anesthetized and perfused with cold PBS followed by a 4% paraformaldehyde solution in PBS. 24 h before, mice received 10 mg/kg of methoxy-X04, i.p., to stain amyloid plaques in the brain parenchyma. Brains were extracted and postfixed overnight in the same fixative. Then, tissues were dehydrated by immersion in a 30% sucrose solution in PBS for 48 h and quickly frozen in cold isopentane. 30 μm-thick floating sections were obtained in a cryostat and stored in anti-freezing solution at – 20 °C.

Floating sections were extensively washed in TBS, pH 7.4, and incubated overnight in a 10% Normal Goat Serum solution (NGS) in TBS. Then, sections were incubated with anti-TREM2 antibody (1:50; MAB17291, clone 237,920, from R&D) in TBS + 10% NGS + 1% Triton X-100, for 24 h. Afterwards, floating sections were extensively washed in TBS and incubated with an Alexa Fluor 546-conjugated secondary antibody (1:100; goat anti-rat IgG (H + L) cross-adsorbed; ref A-11081, from Invitrogen) in TBS/NGS/Triton X-100 for 2 h at 37 °C. Sections were then washed and mounted in glass slides with Vectashield (Vector Laboratories).

Images (eight different fields of brain cortex per mouse) were captured with a Leica Thunder Imager 3D Assay system coupled to a K5 camera and using a 20X/0.40 Plan Fluor objective. Images were processed with ImageJ (NIH, Baltimore, MD, USA) by means of auto-threshold function and subsequent analysis of the binary images. To determine the intensity of TREM2 signal, ROIs of 65 μm diameter were defined around individual amyloid (methoxy-X04 +) plaques and the fluorescence inside them quantified.

### Experimental design and statistical analyses

All statistical analyses were performed and graphs were generated using GraphPad Prism v 6.0 (GraphPad). Graphs represent average values ± standard error of the mean. Only male animals were used in the experiments. The number of animals used for each experiment is reported in the figure legends.

In electrophysiological experiments, statistical differences were calculated according to non-parametric tests. For pairwise comparisons, *p* values were calculated according to two-tailed Mann–Whitney tests (for unpaired data) or Wilcoxon tests (for paired data). For spine morphology and RT-qPCR experiments, statistical analysis were made using two-way analysis of variance (ANOVA) with Tukey’s post-test for multiple comparisons. A *p* value < 0.05 was considered as statistically significant.

## Results

### FAAH genetic inactivation restores basal synaptic transmission in 5xFAD mice

It is well established that 5xFAD mice exhibit cognitive impairment at 6 months of age. We previously reported that the genetic inactivation of FAAH rescues the memory deficit in 5xFAD mice [10]. In this work, we aimed to evaluate the effect of FAAH deletion on neuronal properties.

**Fig. 1:**
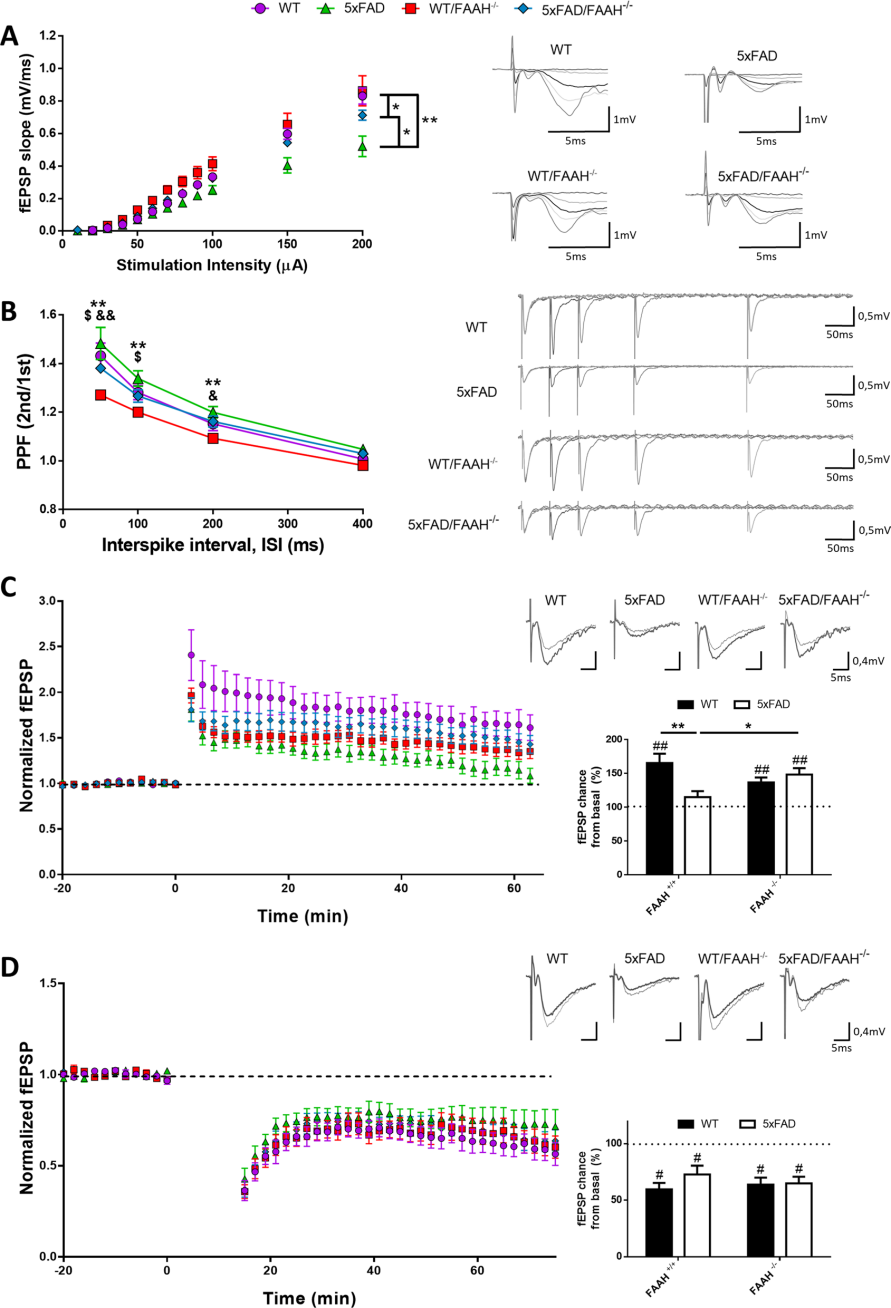
FAAH deletion rescues synaptic plasticity in 5xFAD/FAAH^−/−^ mice.** A** Input/output curves of fEPSPs evoked at the CA3–CA1 synapse by single pulses of increasing intensities in WT (*n* = 11), FAAH^−/−^ (*n* = 13), 5xFAD (*n* = 10), and 5xFAD/FAAH^−/−^ (n = 12) mice (*p < 0.05 and **p < 0.01, according to the Mann–Whitney test). **B** Paired-pulse facilitation ratios from synaptic responses evoked by stimulation of the Schaffer collateral fibres with different interstimulus intervals (**p < 0.01 comparing WT/FAAH^−/−^ vs 5xFAD; ^$^*p* < 0.05 comparing WT/FAAH^−/−^ vs WT; ^&^*p* < 0.05, ^&&^*p* < 0.01 comparing WT/FAAH^−/−^ vs 5xFAD/FAAH^−/−^, according to Mann–Whitney test). **C.** fEPSPs were recorded from CA3-to-CA1 synapses and normalized to the average baseline value before LTP induction. Left, Time course of fEPSPs before and after theta-burst LTP induction from WT (n = 10), FAAH^−/−^ (*n* = 13), 5xFAD (*n* = 10) and 5xFAD/FAAH^−/−^ (*n* = 12) mice. Right, Average responses collected from the last 10 min of the recording and normalized to the baseline. 5xFAD slices failed to display significant potentiation (*p* = 0.18, according to Wilcoxon test). On the contrary, WT, FAAH^−/−^and 5xFAD/FAAH^−/−^ slices were significantly potentiated with respect to their baselines (^##^*p* < 0.01, Wilcoxon test). The Mann–Whitney test was used to compare the extent of potentiation among genotypes (*p < 0.05; **p < 0.01). **D** fEPSPs were recorded from CA3-to-CA1 synapses and normalized to the average baseline value before LTD induction. Left, Time course of fEPSPs before and after induction of NMDA receptor-dependent LTD from WT (n = 8), FAAH^−/−^ (*n* = 9), 5xFAD (*n* = 7) and 5xFAD/FAAH^−/−^ (*n* = 9) mice. Right, Average responses collected from the last 10 min of the recording and normalized to the baseline. WT and FAAH^−/−^ slices were significantly depressed with respect to their baseline, as well as 5xFAD and 5xFAD/FAAH^−/−^ slices (^#^*p* < 0.05; ^##^*p* < 0.01, according to Wilcoxon test). There were no significant differences among genotypes, according to Mann–Whitney test. For all panels, data are presented as means ± s.e.m. and representative traces are shown on the right

Aβ accumulation in the 5xFAD mouse model drives a series of neuropathological alterations that underlie the memory deficit, including a reduction in baseline excitatory transmission at hippocampal CA3-to-CA1 glutamatergic synapses [19]. To assess the effect of FAAH deletion in this context, we first tested basal synaptic properties in CA1 hippocampal neurons from 5 and 5xFAD/FAAH^−/−^ mice. Wild-type and FAAH^−/−^ littermates were used as controls. Acute hippocampal slices were prepared from 6-month-old animals of the four genotypes, and input/output experiments were performed. We measured the slope of the field excitatory postsynaptic potential (fEPSP) recorded from the *stratum radiatum* of CA1, as a function of the stimulation intensity applied to the Schaffer collateral fibres. As shown in Fig. 1A, basal synaptic transmission in CA1 neurons was decreased in 5xFAD mice, as expected. Interestingly, FAAH genetic inactivation partially restored this deficit in 5xFAD/FAAH^−/−^ animals, even though FAAH deletion per se had no effect on basal synaptic function, as compared to WT slices.

Endocannabinoids, including anandamide, work as retrograde messengers and contribute to the modulation of synaptic transmission via presynaptic cannabinoid receptors (for a thorough review, see [20]). Therefore, we asked whether presynaptic properties might be modified in FAAH^−/−^ mice. We tested paired-pulse facilitation (PPF), which reflects the probability of neurotransmitter release. PPF was reduced in FAAH^−/−^ animals, indicating an increased probability of neurotransmitter release at CA3–CA1 synapses (Fig. 1B). On the contrary, PPF was comparable to WT in 5xFAD and 5xFAD/FAAH^−/−^ slices. This result suggests that chronically increased anandamide-dependent endocannabinoid signalling modulates presynaptic function in hippocampal CA3–CA1 synapses; nevertheless, this modulation is absent in the context of amyloid pathology.

### LTP is rescued in 5xFAD/FAAH^−/−^ mice, but LTD is unaffected

Long-term changes in synaptic efficacy, such as long-term potentiation (or LTP), are thought to underlie memory and learning processes [21, 22]. LTP at hippocampal CA3-to-CA1 synapses is significantly reduced in 6-month-old 5xFAD mice [19]. Thus, we asked whether a rescue in LTP deficit might underlie the memory improvement observed in 5xFAD/FAAH^−/−^ animals compared to 5xFAD mice [10]. We used a theta-burst stimulation protocol (TBS: 5 trains, each with 10 bursts at 5 Hz, each burst containing 4 pulses at 100 Hz) to induce LTP of CA3–CA1 excitatory synapses in hippocampal slices from 5 and 5xFAD/FAAH^−/−^ animals, with WT and FAAH^−/−^ slices as controls. As shown in Fig. 1C, WT animals displayed significant synaptic potentiation (65.35 ± 13.77% potentiation, with p = 0.005, according to Wilcoxon test), whereas LTP was impaired in 5xFAD animals, as expected (14.93 ± 8.74% potentiation, with *p* = 0.18, according to Wilcoxon test). In contrast, 5xFAD/FAAH^−/−^ slices yielded significant potentiation (48.28 ± 9.55%, with *p* = 0.006), comparable to that displayed by FAAH^−/−^ mice (36.83 ± 7.05%, with *p* = 0.002).

To further evaluate the impact of FAAH genetic inactivation on synaptic plasticity, we tested long-term depression (LTD) in hippocampal slices from FAAH-null mice, comparing to WT mice. NMDAR-dependent LTD was induced with 1 Hz stimulation for 15 min while recording fEPSP in CA1 *stratum radiatum*. WT and FAAH^−/−^ slices displayed a strong and almost identical depression, as shown in Fig. 1D (40.35 ± 5.68% and 36.13% ± 6.13% depression, with p = 0.01 and p = 0.02, respectively, according to Wilcoxon test). LTD was also preserved in 5xFAD and 5xFAD/FAAH^−/−^ animals (27.16 ± 7.82% and 35.03 ± 5.90% depression, respectively, with p = 0.008 in both cases). No significant difference in synaptic depression was observed among these genotypes, pointing to a lack of effect of FAAH deletion on NMDAR-dependent LTD, both in normal and pathological conditions.

### Synaptic plasticity improvement is not dependent on CB1 or TRPV1 receptor activation

**Fig. 2:**
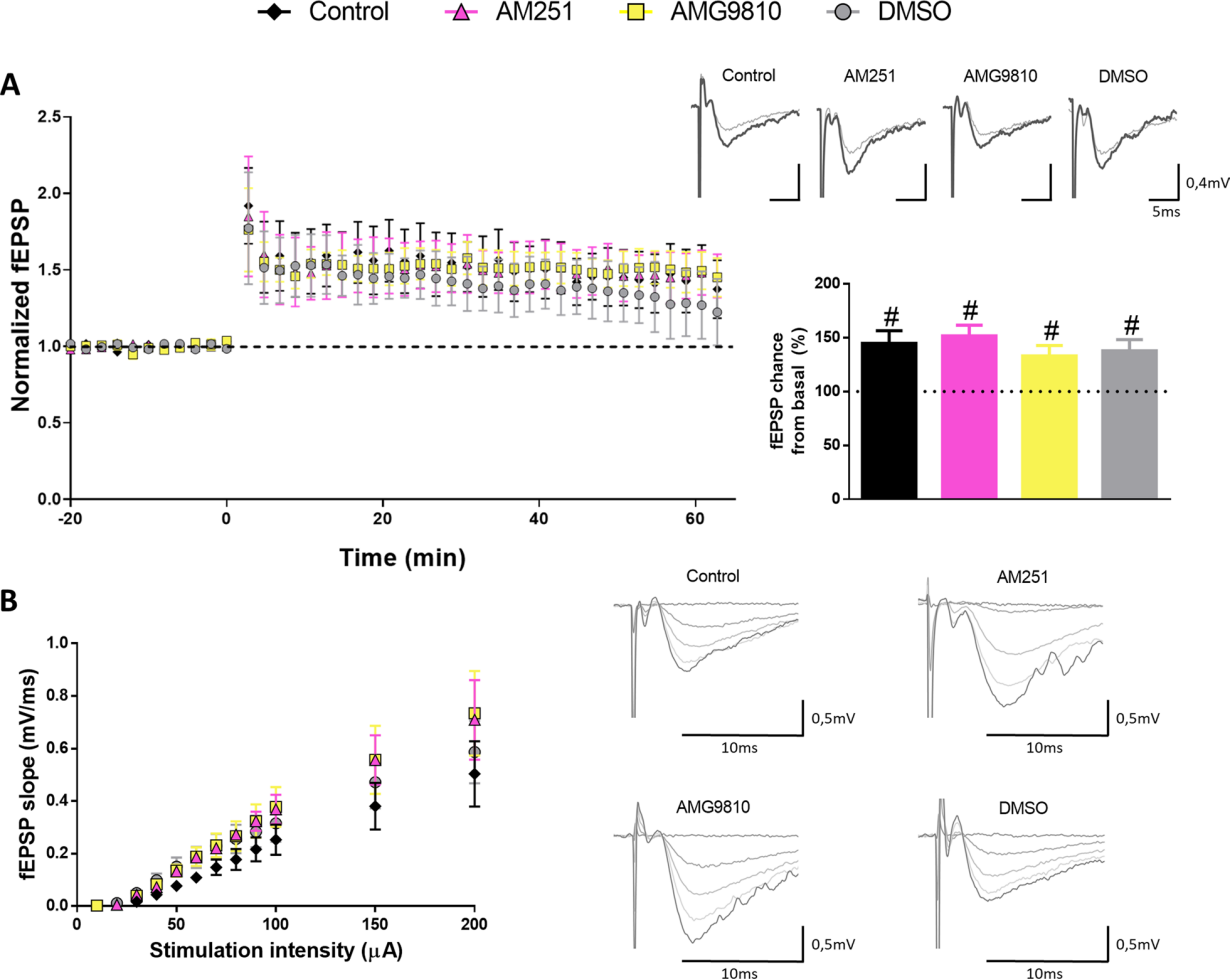
Effect of CB1R or TRPV1 receptor blockade on synaptic function and plasticity in 5xFAD/FAAH^−/−^ mice. Acute slices from 5xFAD/FAAH^−/−^ mice were preincubated with AM251 [4 µM], AMG9810 [3 µM] or DMSO for 20 min. Drugs or vehicle were added to circulating ACSF during recordings as well. **A** fEPSPs were recorded from CA3-to-CA1 synapses and normalized to the average baseline value before LTP induction. Left, Time course of fEPSPs before and after theta-burst LTP induction (5 trains of 10 bursts at 5 Hz each, 1 burst = 4 pulses at 100 Hz) from 5xFAD/FAAH^−/−^ slices treated with AM251 (n = 6 mice), AMG9810 (n = 6 mice), or DMSO (n = 7 mice), using untreated 5xFAD/FAAH^−/−^ slices as controls (n = 7 mice). Right, Average responses collected from the last 10 min of the recording and normalized to the baseline, with representative traces shown above. Wilcoxon tests revealed significant potentiation in both untreated and treated slices (#p < 0.05). **B** Input/output curves of fEPSPs evoked at the CA3–CA1 synapse by single pulses of increasing intensities (10–200 µA) in untreated slices (n = 7 mice), and in slices treated with AM251 (n = 7 mice), AMG9810 (n = 7 mice), or DMSO (n = 10 mice) from 5xFAD/FAAH^−/−^ animals, with representative traces shown on the right. No significant differences among treatment groups were found according to Mann–Whitney tests. For all panels, data are presented as means ± s.e.m

Anandamide binds to and activates CB1Rs, which act as regulators of synaptic transmission and synaptic plasticity in different regions of the brain [23]. Therefore, we tested if LTP rescue in 5xFAD/FAAH^−/−^ animals might be dependent on CB1R activation. To do so, acute hippocampal slices were prepared from 6-month-old 5xFAD/FAAH^−/−^ mice. Slices were either incubated with an antagonist of CB1R, AM251, or with vehicle, prior to and during fEPSP recordings. As shown in Fig. 2A, synaptic transmission at CA3-to-CA1 hippocampal synapses was potentiated in either case (51.70 ± 9.99% potentiation, p = 0.03, and 37.87 ± 10.45% potentiation, *p* = 0.03, respectively). No difference in overall potentiation was observed between slices incubated with the antagonist or with vehicle (*p* = 0.52, according to Mann–Whitney test), indicating that CB1R activation is not necessary to induce and/or maintain LTP in 5xFAD/FAAH^−/−^ animals.

Transient receptor potential vanilloid 1 (TRPV1), which is also a target of anandamide, has been recently implicated in the expression of LTP at hippocampal CA1 region [24]. Thus, we evaluated if TRPV1 antagonism with a specific drug (AMG9810) might reverse the beneficial effect on synaptic plasticity observed in 5xFAD FAAH-null mice. Treatment of slices with AMG9810 (both prior to and during recordings) did not alter LTP, comparing to 5xFAD/FAAH^−/−^ slices incubated with DMSO (33.01 ± 9.85% potentiation, *p* = 0.03, and 37.87 ± 10.45% potentiation, *p* = 0.03, respectively, Fig. 2A). Notably, incubation with AM251 or AMG9810 did not affect basal synaptic transmission either, compared to vehicle (Fig. 2B). These combined results support the idea that CB1R or TRPV1 sustained activation is not responsible for the recovery in synaptic plasticity observed in 5xFAD animals upon FAAH genetic inactivation.

### FAAH deletion normalizes dendritic spine density in 5xFAD mice

**Fig. 3:**
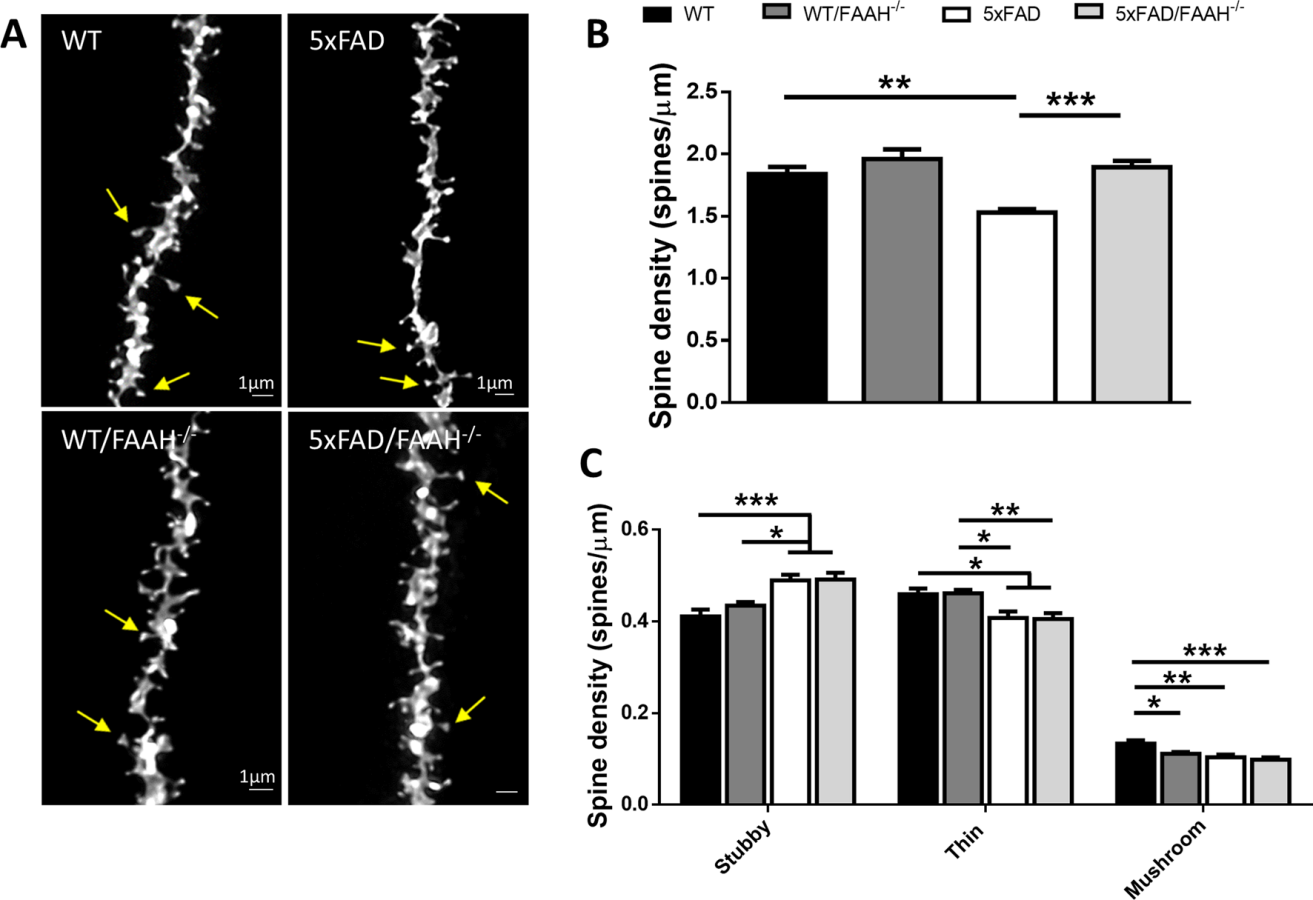
Dendritic spine density and morphology in the hippocampus of WT, FAAH^−/−^, 5xFAD, and 5xFAD/FAAH^−/−^ mice. **A** Deconvoluted confocal images of apical dendrites from DiI-labelled CA1 pyramidal neurons in 6-month-old WT, FAHH^−/−^, 5xFAD, and 5xFAD/FAAH^−/−^ mice. Yellow arrows point to mushroom spines. **B** The dendritic spine density was determined in all four genotypes using the Neuron Studio freeware. Two-way ANOVA followed by Tukey’s test was used to determine differences among groups (**p < 0.01, ***p < 0.001). **C** Density of each type of dendritic spine (stubby, thin, and mushroom) in apical dendrites of CA1 pyramidal neurons in 6-month-old WT, FAHH^−/−^, 5xFAD, and 5xFAD/FAAH^−/−^ mice. Two-way ANOVA followed by Tukey’s test (*p < 0.05, **p < 0.01, ***p < 0.001). For all panels, data are presented as means ± s.e.m. (*n* = 21 dendrites per genotype from 3 to 4 mice per genotype)

Deficits in synaptic function constitute an early event in AD and correlate well with cognitive decline in patients; among those deficits, synapse loss plays a prominent role [25]. Synaptic degeneration has been reported in the 5xFAD mouse model [11], contributing to the spatial memory impairment. In the 5xFAD mouse model, in accordance with the cognitive and synaptic impairments, a reduced density of dendritic spines in cortical and hippocampal neurons has been documented [11, 25]. Therefore, we aimed to confirm this observation by quantifying dendritic spines in hippocampal neurons of 5xFAD animals (Fig. 3A). Indeed, DiOlistic labelling of CA1 principal neurons revealed fewer dendritic spines in apical dendrites of 6-month-old 5xFAD mice compared to age matched WT controls (1.51 ± 0.03 mean spine density in 5xFAD animals vs 1.87 ± 0.06 spines per micron in WT controls, *p* < 0.001).

We next sought to investigate whether FAAH deletion had an impact on the number of dendritic spines in pyramidal neurons. To do so, we labelled CA1 neurons in hippocampal slices from 5xFAD/FAAH^−/−^ mice and quantified spines at apical dendrites (Fig. 3A, B). In 5xFAD/FAAH^−/−^ slices, spine density in secondary dendrites of CA1 pyramidal neurons was equivalent to that found in WT slices and FAAH^−/−^ slices (1.87 ± 0.05, 1.87 ± 0.06 and 1.98 ± 0.08 mean spine density, respectively). Thus, FAAH deletion restores normal spine density in 5xFAD animals.

### The genetic inactivation of FAAH is associated with changes in dendritic spine morphology

Then, we examined dendritic spine morphology in the same pyramidal neurons. As shown in Fig. 3C, we quantified the density of the three different spine types (mushroom, thin, and stubby) in slices from WT, FAAH^−/−^, 5xFAD and 5xFAD/FAAH^−/−^ animals. Stubby spines were markedly increased in 5xFAD animals and 5xFAD/FAAH^−/−^ animals compared to WT and FAAH^−/−^ mice (0.49 ± 0.01 stubby spines per micron for both 5xFAD and 5xFAD/FAAH^−/−^ slices vs 0.41 ± 0.02 and 0.43 ± 0.01 density of stubby spines in WT and FAAH^−/−^ mice, respectively). On the contrary, thin spines were significantly decreased in 5xFAD and 5xFAD/FAAH^−/−^ animals compared to WT mice (0.42 ± 0.01 and 0.41 ± 0.01 density of thin spines, respectively, vs 0.46 ± 0.01 thin spines per micron in 5xFAD animals, with p = 0.02 for both comparisons) and to FAAH^−/−^ mice (0.46 ± 0.01 thin spines per micron in 5xFAD/FAAH^−/−^ animals, with *p* = 0.01 and *p* = 0.009, for the comparison against 5xFAD and 5xFAD/FAAH^−/−^ mice, respectively), while no significant differences were observed between WT and FAAH^−/−^ animals.

Interestingly, the number of mushroom spines was decreased in all three genotypes (0.11 ± 0.003, 0.10 ± 0.006 and 0.10 ± 0.006 density of mushroom spines in FAAH^−/−^, 5xFAD and 5xFAD/FAAH^−/−^ animals, respectively) compared to WT controls (0.13 ± 0.007 mushroom spines per micron, with *p* = 0.04, *p* = 0.003 and *p* = 0.0002, for the comparison against FAAH^−/−^, 5xFAD and 5xFAD/FAAH^−/−^ littermates, respectively). These data show that, although FAAH deletion appears to have an impact on the number of mushroom spines, it does not prevent the changes in spine morphology observed in 5xFAD mice, comparing to WT animals. Therefore, the recovery of spine density in 5xFAD/FAAH^−/−^ mice, as compared to 5xFAD, appears to occur across all spine types, irrespective of their morphology.

### FAAH deficiency enhances microglial activation and amyloid beta phagocytosis

The reversal of LTP impairment and dendritic spine loss in neurons from 5xFAD/FAAH^−/−^ animals demonstrates that FAAH genetic inactivation is associated with a functional and structural recovery of hippocampal neurons in the 5xFAD model of AD. We next sought to investigate the cellular and molecular mechanisms underlying this phenotypic rescue. We previously showed that FAAH deletion drives the increased activation of microglia, the professional phagocytes in brain, together with an overall decrease in amyloid levels and neuritic plaques [10]. It is widely accepted that amyloid beta oligomers impair LTP [26–29] and induce the gradual loss of dendritic spines [30, 31], acting as toxic species that trigger synaptic dysfunction and degeneration prior to neuronal death. The enhanced clearance of Aβ peptides from brain parenchyma would thus prevent their toxic effects on neurons. We hypothesized that activated microglial cells might be responsible for the increased phagocytosis of amyloid beta peptides, and hence their augmented elimination from brain in FAAH-null animals, acting as a protective mechanism against Aβ-induced neuronal damage.

**Fig. 4:**
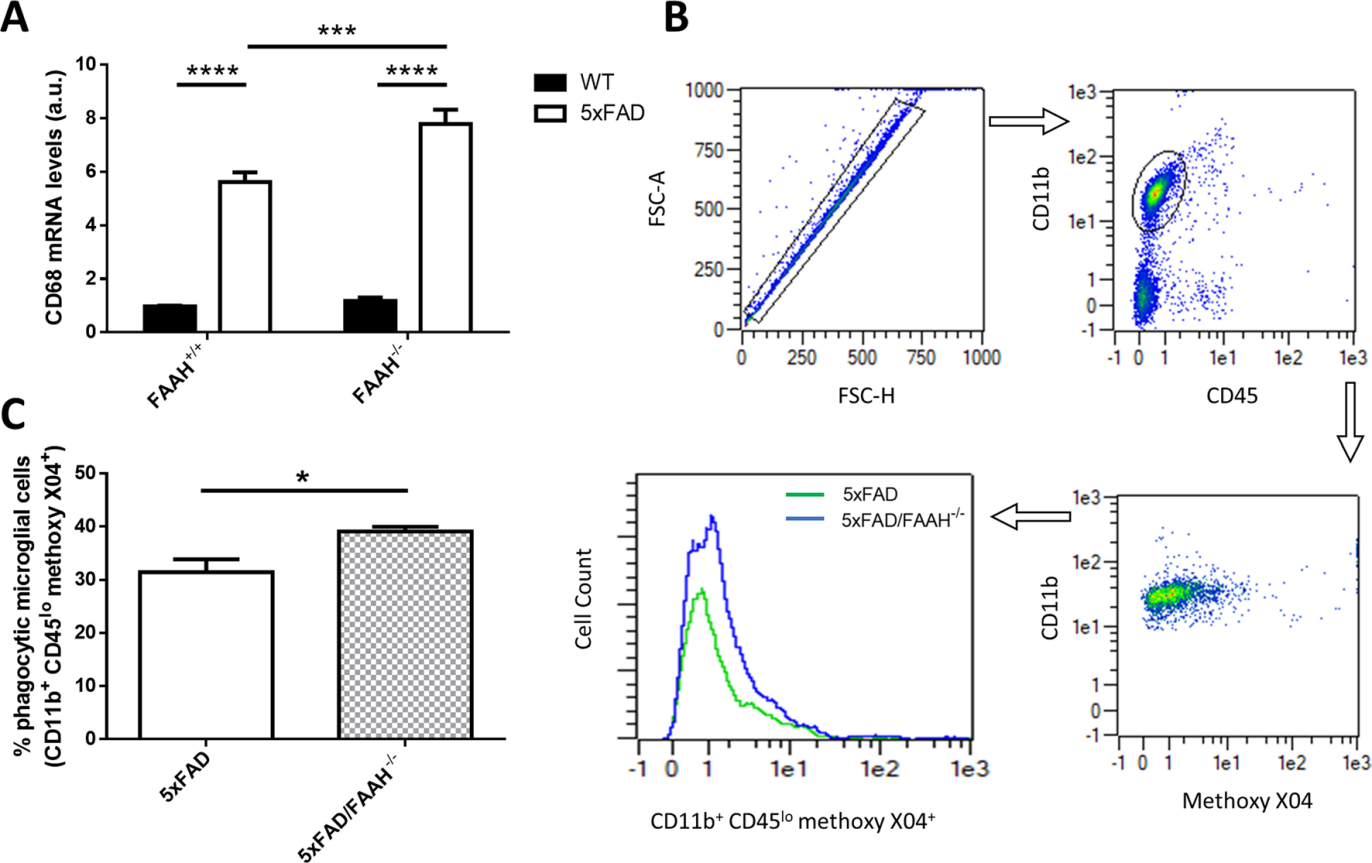
FAAH deletion potentiates activation of microglia and phagocytosis of Aβ in 5xFAD mice. **A** Expression levels of activated microglia marker CD68 obtained from hippocampal extracts after RT-qPCR.** B** Representative flow plots of the gating strategy for identification of phagocytic microglia cells (CD11b + CD45 low) isolated from 5 and 5xFAD/FAAH^−/−^ mice. **C** Quantification of phagocytic microglial cells (expressed as the percentage of total singlet cells that are CD11 + , CD45lo, Methoxy X04 + cells) in 5xFAD and 5xFAD/FAAH^−/−^ mice. Statistical analysis: for **A**, two-way ANOVA followed by Tukey’s test (***p < 0.001, ****p < 0.0001) (*n* = 7 animals in each group); for **C**, unpaired t test with Welch’s correction (*p* = 0.04) (*n* = 4 animals in each group)

First, we verified the activation state of microglial cells in 5xFAD animals when FAAH is genetically inactivated. CD68 is a phenotypic marker known to be upregulated in activated microglia [32]. As shown in Fig. 4A, CD68 mRNA levels were increased in 5xFAD animals comparing to WT counterparts (p < 0.0001), but are even further elevated in 5xFAD/FAAH^−/−^mice (with p = 0.0005 for the comparison between 5 and 5xFAD/FAAH^−/−^ littermates), thus confirming our previous observation [10].

We next evaluated whether amyloid beta phagocytosis is enhanced upon FAAH deletion in the 5xFAD mouse model. We injected methoxy X04, that specifically labels Aβ aggregates, to 6-month-old 5xFAD and 5xFAD/FAAH^−/−^ animals and measured microglial phagocytic activity by flow cytometry. As shown in Fig. 4B, C, a significant increase in the percentage of phagocytic microglia (CD11b positive, CD45 low, methoxy X04 positive) was observed in 5xFAD/FAAH^−/−^ animals, as compared to 5xFAD (39.11 ± 0.88% and 31.45 ± 2.41% of phagocytic microglia, respectively; p = 0.04). These results suggest that the increased endocannabinoid tone in 5xFAD/FAAH^−/−^ animals might potentiate the ability of microglia to clear Aβ, thus contributing to preserve neuronal structure and functionality in the AD brain.

### TREM2 is specifically upregulated in the brains of 5xFAD/FAAH^−/−^ mice compared to 5xFAD animals

**Fig. 5:**
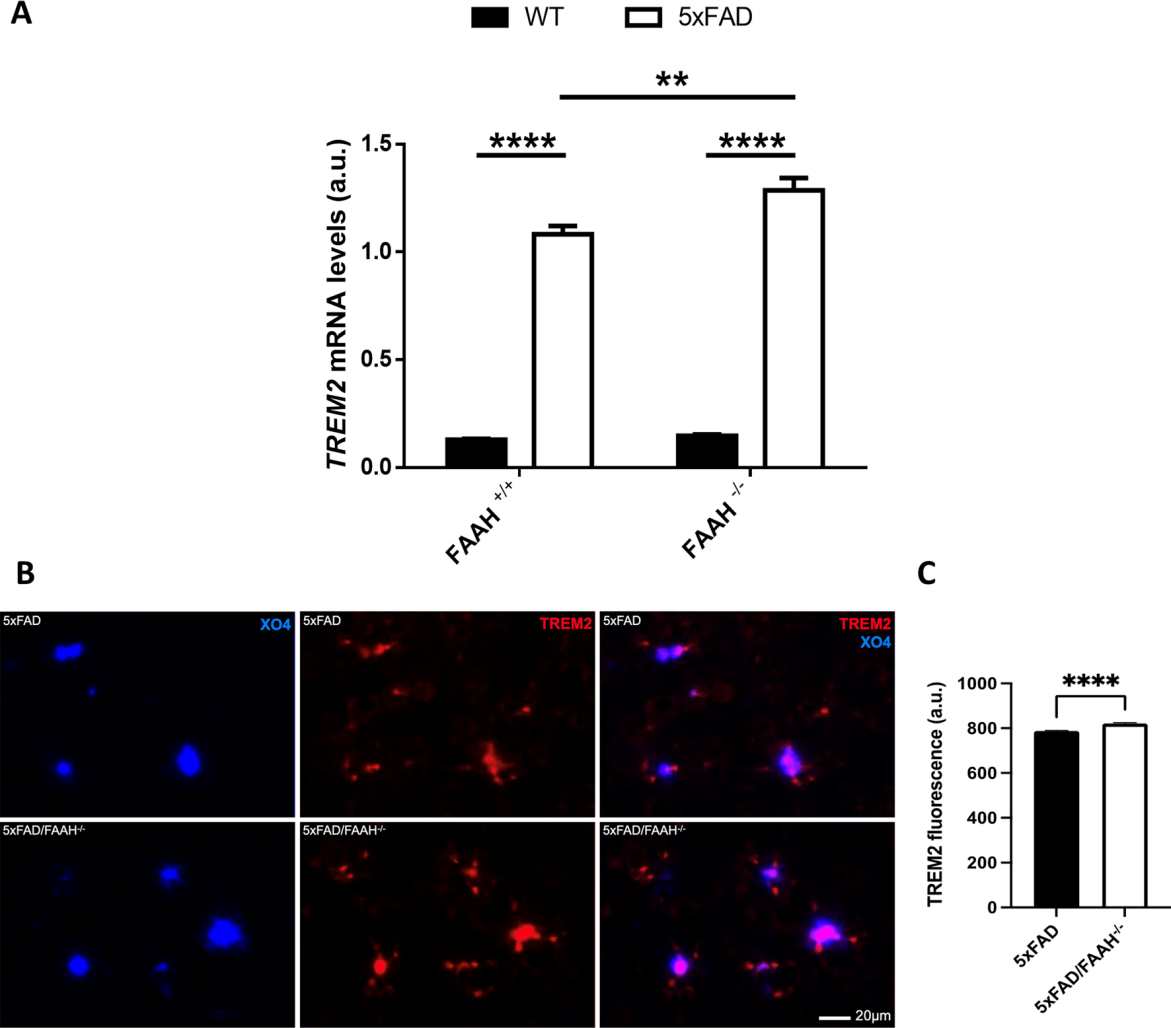
FAAH deletion enhances the expression of TREM2 in 5xFAD mice.** A** Expression levels of TREM2 (**A**), obtained from hippocampal extracts after RT-qPCR. Two-way ANOVA followed by Tukey’s test (**p < 0.01, ****p < 0.0001) (*n* = 6–8 animals in each group). Graphs represent mean ± s.e.m. **B** Representative images showing TREM2 protein expression in microglia surrounding amyloid plaques in cortex, as revealed by immunofluorescence. Blue channel (left) corresponds to amyloid plaque labelling with methoxy-XO4; red channel (middle) shows anti-TREM2 staining. Merged images are shown in the right panels. **C** Densitometric quantification of TREM2 fluorescence. N = 3 mice per group. ****p < 0.0001 by Mann–Whitney test. Error bars represent mean ± s.e.m. Scale bars, 20 μm

To further characterize the microglial involvement in the phenotype of 5xFAD/FAAH^−/−^ mice, we analysed microglial markers associated with phagocytosis that have been shown to play a relevant role in AD. Mutations in TREM2 (triggering receptor expressed on myeloid cells 2) have been identified as risk factors for AD in genetic studies [33, 34]. TREM2 is an innate immune receptor preferably expressed by microglia that stimulates phagocytosis of Aβ and apoptotic neurons [35–37]. Therefore, we assessed if changes in TREM2 expression might underlie the increased phagocytic activity of microglia in 5xFAD/FAAH^−/−^ animals. As shown in Fig. 5A, TREM2 mRNA levels were increased in 5xFAD animals, as expected (with *p* < 0.0001 for the comparison between WT and 5xFAD, and the comparison between FAAH^−/−^ and 5xFAD/FAAH^−/−^ mice). Remarkably, FAAH genetic inactivation was associated with an even higher enhancement of expression in pathological conditions (with *p* = 0.005 for the comparison between 5 and 5xFAD/FAAH^−/−^ littermates).

We then examined TREM2 protein levels in microglia surrounding amyloid plaques by immunostaining. As shown in Fig. 5B, TREM2 expression was enhanced in microglia clustered around amyloid plaques in brain slices from 5xFAD/FAAH^−/−^ animals comparing to 5xFAD mice. This increase was significant (Fig. 5C), thus strengthening the result obtained by RT-qPCR.

**Fig. 6:**
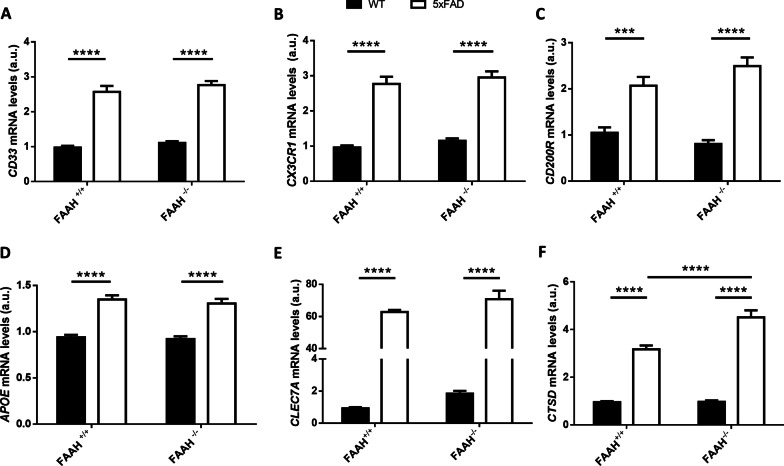
Mixed DAM signature in microglia from 5xFAD/FAAH^−/−^ mice.** A**–**C** Expression levels of phagocytic markers CD33 (**A**), CX3CR1 (**B**) and CD200R (**C**), obtained from hippocampal extracts after RT-qPCR. **E**–**G** Messenger RNA levels of DAM markers APOE (**D**), CLEC7A (**E**) and CTSD (**F**), obtained from hippocampal extracts after RT-qPCR. Two-way ANOVA followed by Tukey’s test (**p < 0.01, ****p < 0.0001) (*n* = 6–8 animals in each group). Graphs represent mean ± s.e.m

Next, we tested whether TREM2 upregulation in 5xFAD FAAH-null mice was specific or whether it reflected a generalized overexpression of microglial surface receptors. CD33 has been identified as a disease risk gene in AD as well, with a negative impact on Aβ microglial uptake [38]. Indeed, recent reports highlight a crosstalk between TREM2 and CD33, which exhibit opposite effects on amyloid plaque burden and microglial activation [39]. As shown in Fig. 6A, we quantified the expression levels of CD33 by reverse-transcriptase quantitative PCR (RT-qPCR) in hippocampal extracts from WT, 5xFAD, FAAH^−/−^ and 5xFAD/FAAH^−/−^ animals. As expected, CD33 expression was increased in samples from 5 and 5xFAD/FAAH^−/−^ mice (with *p* < 0.001 for the comparison against WT and FAAH^−/−^ mice, respectively), but this increment was independent of FAAH deletion (*p* = 0.65 when comparing 5xFAD and 5xFAD/FAAH^−/−^ mice).

Finally, we quantified the expression levels of CX3CR1 and CD200R (Fig. 6B, C), two microglial receptors that have also been linked to Aβ deposition and clearance by microglia in AD models, and have been shown to mediate neuron–glia communication [40]. CX3CR1 deficiency results in a gene dose-dependent reduction in β-amyloid deposition and increased phagocytic activity in AD mouse models [41, 42]. On the other hand, microglial activation has been demonstrated to depend on the interaction between neuronal CD200 and its receptor, CD200R, which is expressed on microglia in the brain. Recent reports have suggested that signalling through the CD200–CD200R pair alters microglial sensitivity to Aβ and its phagocytic activity, although with contradictory results [43, 44]. As shown in Fig. 6B, C, CX3CR1 and CD200R expression was enhanced in the presence of the 5xFAD transgenes (with p = 0.0003 for the comparison between 5xFAD and WT, and *p* < 0.0001 for the comparison between 5xFAD/FAAH^−/−^ and FAAH^−/−^ animals) but unaffected upon FAAH genetic inactivation. In summary, these data reveal that, although 5xFAD mice display increased expression of several microglial receptors, only TREM2 is specifically upregulated upon FAAH genetic inactivation in pathological conditions.

### Microglia from 5xFAD/FAAH^−/−^ mice exhibit a mixed DAM signature

Some of the above mentioned genes are either downregulated or upregulated in disease-associated microglia (DAM), a specific subpopulation of microglia recently identified in AD mouse models and human patients, and seemingly associated with restricting disease progression [45]. DAM cells display phagocytic activity and cluster around Aβ plaques [45]. We, therefore, decided to proceed with the phenotypic characterization of microglia in the 5xFAD/FAAH^−/−^ mouse model, specifically interrogating DAM markers that have been identified as AD risk genes, such as APOE [46] and CTSD [47], and related to microglial phagocytic activity, such as CLEC7A [48], as well. We also analysed marker genes of homeostatic microglia, such as P2RY12 and TMEM119, that have been shown to be downregulated in DAM microglia [45]. We performed RT-qPCR experiments to measure mRNA levels of each of these markers in WT groups (WT and FAAH^−/−^) and AD animals (5xFAD and 5xFAD/FAAH^−/−^). The expression of microglial homeostatic genes was unchanged between 5 and 5xFAD/FAAH^−/−^ mice (Additional file 1: Fig. S1). As shown in Fig. 6D–F, the expression of the three markers related to phagocytosis (APOE, CLEC7A and CTSD) was enhanced in AD animals comparing to WT groups. Remarkably, only CSTD was specifically upregulated in 5xFAD/FAAH^−/−^ mice comparing to 5xFAD littermates (with *p* < 0.0001 for the comparison between these two groups). These data suggest a specific gene expression profile related to DAM molecular signature in microglia from 5xFAD/FAAH^−/−^ mice.

### Complement component C3 and its receptor C3AR are specifically upregulated in the brains of 5xFAD/FAAH^−/−^ mice compared to 5xFAD animals

**Fig. 7:**
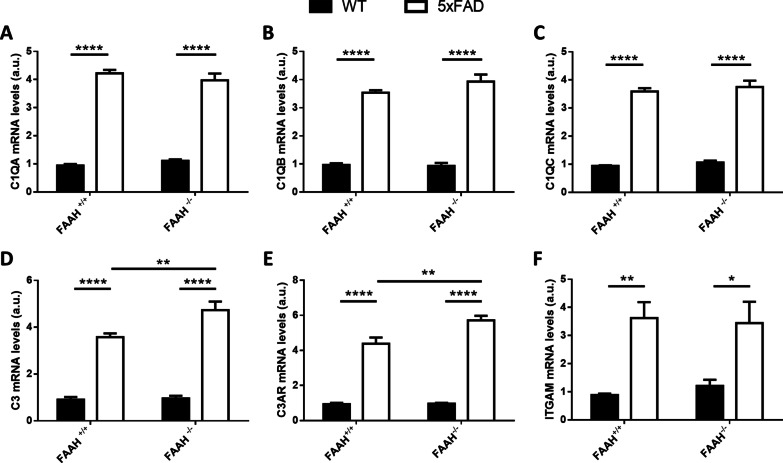
FAAH deletion enhances the expression of C3 and C3AR in 5xFAD mice. **A**–**C** Expression levels of complement C1QA (**A**), C1QB (**B**), C1QC (**C**), from hippocampal extracts after RT-qPCR. **D**–**F** Messenger RNA levels of complement component C3 (**D**) and its receptors C3AR (**E**) and ITGAM (**F**), obtained from hippocampal extracts after RT-qPCR. Two-way ANOVA followed by Tukey’s test (*p < 0.05, **p < 0.01, ****p < 0.0001) (*n* = 6–8 animals in each group). Graphs represent mean ± s.e.m

According to our data, Aβ enhanced clearance seems to play a predominant role in the prevention of neuronal damage in the 5xFAD FAAH-null model. Thus, we aimed to deepen the characterization of molecular players related to Aβ phagocytosis by microglia in 5xFAD/FAAH^−/−^ animals, by evaluating components of the complement cascade.. Complement components have been reported to either modulate the phagocytosis of Aβ by microglia, in the case of C1Q [49], or promote it, in studies evaluating the contribution of C3 to plaque clearance. [49, 50]. Nevertheless, recent evidence points to a deleterious effect of these mediators on synapse elimination, as inhibition or knockout of C1Q, or C3 reduced synapse loss in AD mouse models [51–53]. To clarify the possible contribution of complement components to the pathophysiological mechanisms operating in the 5xFAD/FAAH^−/−^ model, we quantified C1QA, C1QB, C1QC and C3 expression levels by RT-PCR in hippocampal extracts from WT, FAAH^−/−^, 5xFAD and 5xFAD/FAAH^−/−^ animals. Figure 7A–C shows that C1Q expression levels were increased as a consequence of amyloidosis but unaffected by FAAH deletion. Strikingly, C3 expression was also augmented in the 5xFAD model, but its mRNA levels were even further increased if FAAH was genetically inactivated in these animals (Fig. 7D,  p=0.0052 for the comparison between 5 and 5xFAD/FAAH^−/−^ mice).

We, therefore, decided to examine the expression of C3 receptors, the C3AR receptor and the C3R receptor, also known as ITGAM, in hippocampal extracts from animals of the four genotypes. It is important to note that C3AR is present in neurons, astrocytes and microglia [54–58], while ITGAM is exclusively expressed by microglial cells [54]. As shown in Fig. 7E, F, both receptors were overexpressed in the presence of the 5xFAD transgenes, while only C3AR expression was further elevated in 5xFAD/FAAH^−/−^ mice, comparing to 5xFAD littermates (with *p* = 0,0019 for the comparison between these two groups). These data would suggest the putative association of C3 complement factor increased expression with the phenotypic improvement associated to FAAH deletion in the 5xFAD mouse model. It also suggests the possible implication of neurons, astrocytes, and microglia in a multicellular response to CNS damage, in which the enhanced endocannabinoid tone would act as a triggering stimulus.

## Discussion

Here, we show that the chronic enhancement of the endocannabinoid tone in an AD mouse model prevents the neuropathology associated to the disorder, possibly through the modulation of microglial ability to phagocytose Aβ. Specifically, FAAH genetic inactivation in 5xFAD mice results in: (1) the restoration of hippocampal synaptic plasticity, (2) the normalization of dendritic spine density in CA1 pyramidal cells, (3) an enhanced uptake of Aβ by microglia, and (4) a selective TREM2, CTSD, C3 and C3AR upregulation, at significant higher levels than those observed in 5xFAD mice.

Synaptic dysfunction and loss preceding neurodegeneration are hallmarks of neuropathology in AD. Nevertheless, the mechanisms leading to synaptic failure in this complex disorder are only partially elucidated. Endocannabinoids have long been recognized as key modulators of synaptic transmission and plasticity in the rodent CNS [20, 23, 59]. Markedly, we found a beneficial effect of the increased endocannabinoid tone in the context of AD pathology. In this study, we report that basal transmission and LTP are rescued in 5xFAD/FAAH^−/−^ animals, and that this effect is independent of CB1R or TRPV1 receptor activation.

The precise involvement of the CB1R in the modulation of synaptic plasticity at hippocampal synapses is still far from clear. Markedly, recent reports suggest a dual effect of CB1R activation on LTP that depends on its specific location at hippocampal synapses. Likewise, opposite effects on dendritic spine density have been documented [60]. In our hands, CB1R blockade did not have an impact on synaptic transmission at CA3-to-CA1 synapses in 5xFAD/FAAH^−/−^ mice. This lack of effect might be a consequence of either the methodological conditions used for these experiments, based on the acute application of the drug on hippocampal slices, or the impossibility to dissect the effects of CB1R antagonism at specific synapses with a pharmacological tool. In any case, it highlights the fact that the phenotypic rescue we now document in 5xFAD/FAAH^−/−^ animals is not mediated by CB1Rs, which is consistent with our previous observations [10].

One could then suggest that other receptors, such as TRPV1 receptors, might be responsible for this effect. We did not observe an impact on LTP when blocking TRPV1 receptors on hippocampal slices from 5xFAD/FAAH^−/−^ animals. This result would corroborate the observation that TRPV1-mediated effects on hippocampal LTP are dependent on GABAergic activation [61], as we use the GABA_A_ antagonist picrotoxin in our recordings. Altogether, these findings suggest that the synaptic deficit amelioration that is observed upon FAAH genetic inactivation in 5xFAD mice is not driven by the activation of canonical AEA receptors.

Noteworthy, a structural recovery was also observed in pyramidal neurons from 5xFAD/FAAH^−/−^ mice. In these animals, a normalization of dendritic spine density was evidenced in CA1 neurons, together with the LTP rescue. We think that this overall neuroprotective effect might be a direct consequence of the reduced accumulation of Aβ peptides in brain. It is widely accepted that soluble oligomers of Aβ peptide not only inhibit LTP in hippocampus [26–29], but also induce synapse and spine loss [30, 31]. In 5xFAD/FAAH^−/−^ mice, the reduced amyloid levels and plaque load we previously documented might be the result of a diminished production of Aβ (via decreased APP expression (10)) and an increased clearance of the toxic species, through the potentiation of microglial phagocytic activity that we now report.

Microglial phagocytic activity is known to be regulated by several transmembrane receptors. In this work, we assessed the levels of expression of CD33, CX3CR1, CD200R and TREM2. Out of the four markers we analyzed, TREM2 was the only one with a differential pattern of expression associated to FAAH deletion. As previously documented, TREM2 was notably upregulated in the AD context [36, 62, 63], but strikingly, its expression was even higher in 5xFAD FAAH-null animals. Remarkably, we now demonstrate that TREM2 protein expression is specifically upregulated in microglia surrounding amyloid plaques in these animals.

TREM2, expressed by microglia in brain [64], is currently thought to be pivotal for microglial response, especially in pathological conditions. In the 5xFAD model of amyloidosis, microglia without TREM2 internalized less fibrillar Aβ [65, 66]. Consequently, the chronic, antibody-mediated activation of TREM2, as well as TREM2 upregulation, resulted in recruitment of microglia to plaques, decreased amyloid deposition and improvement in spatial learning and novel object recognition memory in AD models [36, 67]. Interestingly, recent data point to the association between TREM2 expression and the appearance of a specific microglial type (“disease-associated microglia”, DAM), with a characteristic molecular signature, in neurodegenerative diseases including AD. DAM was first shown to restrict disease progression by Keren-Shaul and colleagues [45]. Their study also revealed that some of the overexpressed genes in DAM are dependent on TREM2 for their upregulation. In a subsequent work, Lee et al. observed that increasing TREM2 gene dosage triggers a transcriptional “reprogramming” that promotes beneficial aspects of microglial function in the AD mouse brain, such as Aβ phagocytosis and suppression of over-activation of the innate immune response [68].

Therefore, a plausible hypothesis is that through the modulation of microglial functions, TREM2 contributes to the reduction in Aβ deposition and consequent loss of synapsis that is observed in AD mouse models [67]. The interpretation that upregulation of TREM2 acts as a compensatory response to Aβ and, through the fine-tuning of microglial response, protects against disease progression, fits nicely with our data. In addition, this neuroprotective effect of microglia activation might be enhanced by a putative role of TREM2 in neuronal survival and neurogenesis, as has been documented in the APP/PS1 transgenic mouse model [62].

Interestingly, microglia from 5xFAD/FAAH^−/−^ brains did not completely recapitulate the DAM signature. In DAM, homeostatic genes (CX3CR1, P2RY12, TMEM119) are downregulated, while the expression of phagocytic pathway genes (TREM2, CLEC7A, CTSD) is enhanced [45]. We did not observe the downregulation of homeostatic genes in AD groups, and while TREM2, CLEC7A and CTSD were indeed overexpressed in the presence of amyloidosis, only TREM2 and CTSD upregulation seemed to be specific of FAAH deletion in pathological conditions. It is important to note that we did not perform single-cell RNA sequencing experiments, hence the reported gene expression profile might reflect the mixed contribution of different microglial phenotypes, maybe at different transition states from homeostatic to DAM. In any case, our results might point to the presence of a DAM-enriched microglial population in 5xFAD/FAAH^−/−^ brains, specifically overexpressing phagocytic-related receptors, such as TREM2 and CTSD. This interpretation would favor the notion that the induction of the DAM program does have a beneficial impact in the context of amyloidosis.

How would the putative increase in TREM2-dependent signaling fit with the pro-inflammatory milieu we have previously reported in 5xFAD FAAH-null animals [10]? The relationship between TREM2 and neuroinflammation is still far from clear. An interesting controversy exists around the presumed anti-inflammatory role of TREM2-mediated signaling. Although TREM2 might play an anti-inflammatory role in certain contexts, numerous studies support the notion that TREM2 can amplify or promote inflammatory responses (for a review, see [69]). It is important to note that FAAH KO astrocytes were identified as the main source of pro-inflammatory cytokines when exposed to amyloid beta in vitro [70]. In the 5xFAD/FAAH^−/−^ mouse model, the increased expression of pro-inflammatory cytokines was CB1R-dependent; on the contrary, the phenotypic rescue we now describe seems to be unrelated to CB1R activation. Although did not directly test if TREM2 expression varies upon CB1R activation, it is tempting to speculate that this apparent discrepancy might well reflect the multifaceted mechanisms of action of endocannabinoids.

In this conceptual framework, an important question remains to be answered. What is the causal link between FAAH deletion and TREM2 overexpression in the presence of amyloid pathology? TREM2 ligands are not well characterized yet. It is known that lipids from cell membranes and lipoprotein complexes can bind and activate TREM2 [71, 72]. Those lipids may accumulate or be released as a consequence of brain damage. Indeed, Wang and colleagues showed that TREM2 acts a sensor for a wide array of lipids associated with Aβ accumulation and neuronal loss in 5xFAD mice [73]. In our model, a significant increase in brain levels of AEA, palmitoylethanolamide and oleoylethanolamide is observed upon FAAH genetic inactivation [10]. Given their lipidic nature, we hypothesize that one or several of these N-acylethanolamines may act as TREM2 ligands, or even induce its expression in microglia.

. In any case, the differential upregulation of TREM2 in 5xFAD/FAAH^−/−^ animals we now demonstrate provides a strong evidence for a potent microglial response. Apart from TREM2 activation by endocannabinoids, another possible explanation is that pro-inflammatory cytokines released by astrocytes directly activate microglia and consequently promote TREM2 expression. This would imply that both astrocytes and microglia are highly sensitive to the chronic increase of endocannabinoids in brain, and that extracellular signals released by astrocytes contribute to a shift in the microglial phenotype. In previous works, we demonstrated that astrocytes and microglia display an exacerbated responsiveness upon FAAH deletion in the 5XFAD model [10, 12], and that in vivo, the ATP released through astrocytic hemichannels was critical for microglial response [12].

Similarly, the increased expression of C3, which is produced by both microglia and astrocytes [74, 75], suggests a possible involvement of astrocytes in a multicellular response against damage. FAAH-KO astrocytes display enhanced NFκB activation [70], which has been recently shown to drive the release of complement C3 [54, 76]. According to these reports, C3 from astrocytes influences dendritic morphology and Aβ phagocytosis through C3AR binding in neurons and microglia, respectively. The C3AR receptor is expressed by neurons, microglia and astrocytes [54–58]. Interestingly, we now show an upregulation of C3AR in 5xFAD mice, that is further increased upon FAAH genetic inactivation. This specific upregulation of C3 and C3AR would imply that the activation of the C3–C3AR axis is required to mediate a multicellular crosstalk in the AD pathological context, in which astrocytes and neurons might orchestrate the microglial response. The potentiation of this response, associated to the enhanced endocannabinoid tone, seems to be protective. As a matter of fact, the idea of an intricate cross-talk between neurons and immune cells to maintain brain homeostasis is not new, and is recently receiving considerable attention [77, 78].

The role of complement activation in AD remains controversial, as well. Several reports point to a detrimental effect of C3 upregulation in AD models [53, 54, 79, 80], while others highlight its ability to potentiate Aβ phagocytosis and clearance of neuritic plaques [50, 81]. This discrepancy might be due to differences in the AD models used and, more importantly, the disease phase in which these contributions are analyzed [82]. It is possible that different mechanisms operate at early vs late stages of AD. In our hands, C3 upregulation, together with TREM2 overexpression, is associated to preserved neuronal function and morphology in 6-month-old 5xFAD/FAAH^−/−^ animals.

## Conclusions

In this work, we demonstrate the potential of the ECS to prevent the neuropathology associated to AD. When the endocannabinoid tone was chronically elevated in brain, a rescue in hippocampal synaptic plasticity at the functional and structural levels was observed in 5xFAD animals. This phenotypic rescue was linked to increased microglial activation, as evidenced by CD68, TREM2 and CTSD specific upregulation in 5xFAD FAAH-null mice. Apart from neurons and microglia, the direct involvement of astrocytes is also suspected, as C3 and C3AR were also specifically upregulated in these mice. In sum, based on the findings we report in this work, we envision a complex scenario in which interactions between astrocytes, microglia, and neurons, driven by endocannabinoids, exert a beneficial effect on AD-associated neuropathology. Via their multiple targets, endocannabinoids might enhance endogenous mechanisms of protection against injury through a multi-faceted modulation of disease progression. Remarkably, our work contributes to the body of evidence that points to the selective inhibition of FAAH as a promising therapeutic strategy in neurodegenerative diseases. To note, we have previously evidenced different effects upon pharmacological and genetic inhibition of FAAH enzyme in this model of AD [10, 12], which we think might be a consequence of long-term adaptations in the FAAH KO mouse. Consequently, a stable, durable inhibition of FAAH activity might be considered as a therapeutic approach to exploit the benefits of ECS modulation in AD.

## Supplementary Information

**Additional file 1: Figure S1. **Homeostatic microglial genes expression in hippocampus of WT, FAAH^−/−^, 5xFAD, and 5xFAD/FAAH^−/−^ mice. Messenger RNA levels of homeostatic microglial markers TMEM119 **A** and P2RY12 **B** obtained from hippocampus extracts after RT-qPCR. Two-way ANOVA followed by Tukey’s test (**p < 0.01, ****p < 0.0001) (*n* = 6 to 8 animals in each group). Graphs represent mean ± s.e.m.

## Data Availability

The data sets generated for this study are available on request to the corresponding authors.

## Abbreviations

5xFAD: Mice co-expressing five familial Alzheimer’s disease mutations
Aβ: Amyloid-β
ACSF: Artificial cerebrospinal fluid
AD: Alzheimer’s disease
AEA: Anandamide
APOE: Apolipoprotein E
APP: Amyloid precursor protein
BSA: Bovine serum albumin
C3AR: Complement C3a receptor 1
CB1R: Cannabinoid 1 receptor
CD200R: CD200 receptor 1
CD33: Cluster of differentiation 33
CD68: Cluster of differentiation 68
CLEC7A: C-type lectin domain containing 7A
CTSD: Cathepsin D
CX3CR1: CX3C chemokine receptor 1
DAM: Disease-associated microglia
DMSO: Dimethyl sulfoxide
ECS: Endocannabinoid system
FAAH: Fatty acid amide hydrolase
fEPSP: Field excitatory postsynaptic potential
GABA: γ-Aminobutyric acid
ITGAM: Integrin subunit alpha M
LTP: Long-term potentiation
LTD: Long-term depression
PBS: Phosphate buffered saline
PPF: Paired-pulse facilitation; P2RY12: purinergic receptor P2Y12
P2RY12: Purinergic receptor P2Y12
RT-qPCR: Reverse-transcriptase quantitative polymerase chain reaction
TMEM119: Transmembrane protein 119
TREM2: Triggering receptor expressed on myeloid cells 2
TRPV1: Transient receptor potential cation channel subfamily V member 1
WT: Wild type
