# Supplementary figures and images for: Potentiation of amyloid beta phagocytosis and amelioration of synaptic dysfunction upon FAAH deletion in a mouse model of Alzheimer's disease
Source: J Neuroinflammation. 2021 Sep 29;18:223. doi: 10.1186/s12974-021-02276-y (PMC8482614; doi:10.1186/s12974-021-02276-y)

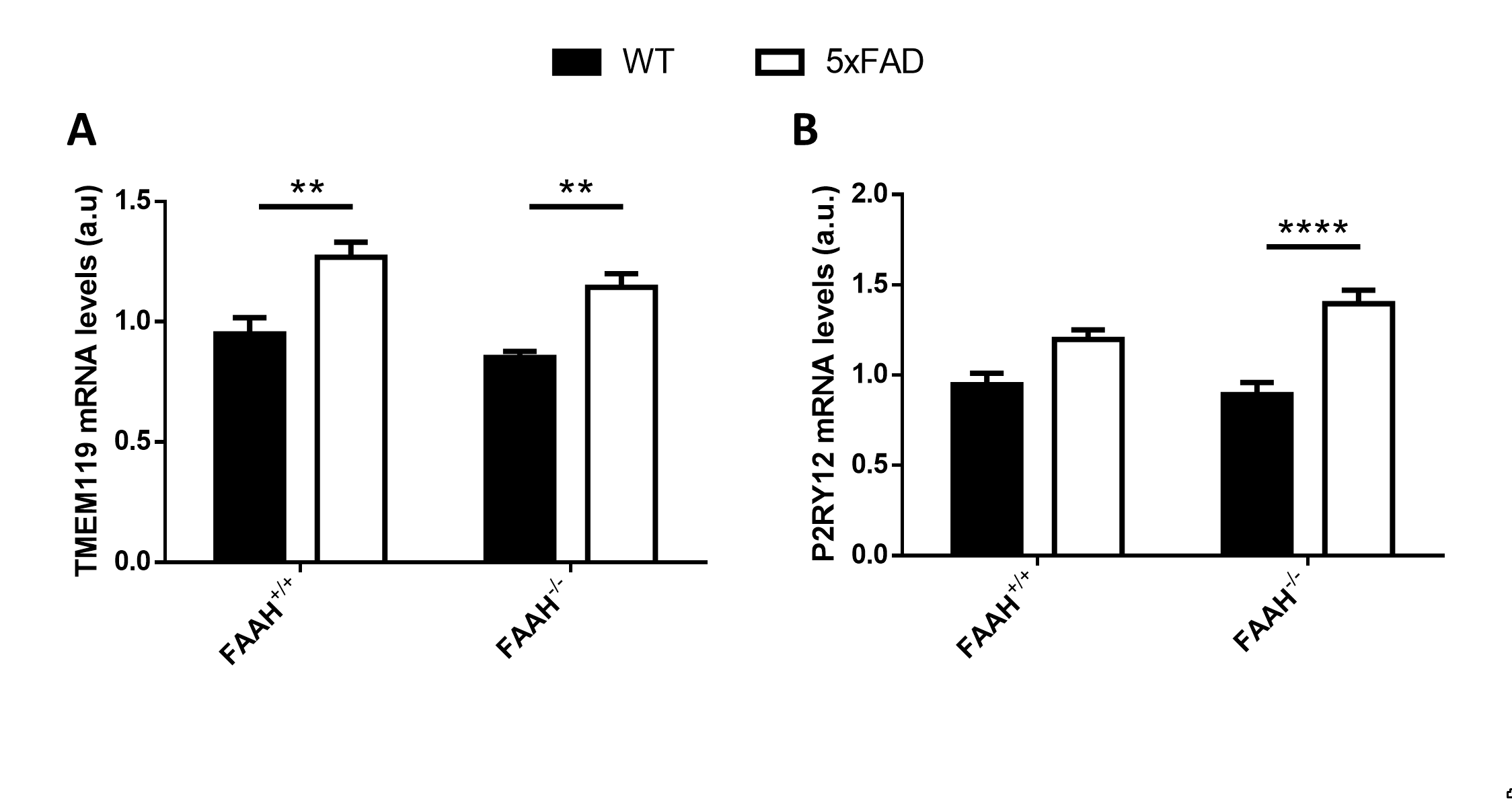

Supplement: Supplementary file 1 — Additional file 1: Figure S1. Homeostatic microglial genes expression in hippocampus of WT, FAAH−/−, 5xFAD, and 5xFAD/FAAH−/− mice. Messenger RNA levels of homeostatic microglial markers TMEM119 A and P2RY12 B obtained from hippocampus extracts after RT-qPCR. Two-way ANOVA followed by Tukey’s test (**p < 0.01, ****p < 0.0001) (n = 6 to 8 animals in each group). Graphs represent mean ± s.e.m. [file 12974_2021_2276_MOESM1_ESM.tif]
